# Conduction system pacing for cardiac resynchronization therapy: State of the art, current controversies, and future perspectives

**DOI:** 10.3389/fphys.2023.1124195

**Published:** 2023-01-13

**Authors:** Edoardo Bressi, Domenico Grieco, Justin Luermans, Haran Burri, Kevin Vernooy

**Affiliations:** ^1^ Department of Cardiology, Cardiovascular Research Institute Maastricht (CARIM), Maastricht University Medical Center, Maastricht, Netherlands; ^2^ Department of Cardiovascular Sciences, Policlinico Casilino of Rome, Rome, Italy; ^3^ Department of Cardiology, University Hospital of Geneva, Geneva, Switzerland

**Keywords:** his bundle pacing, left bundle branch area pacing, heart failure, left bundle branch block, biventricular pacing, conduction system pacing

## Abstract

Biventricular pacing (BVP) is the established treatment to perform cardiac resynchronization therapy (CRT) in patients with heart failure (HF) and left bundle branch block (LBBB). However, BVP is an unnatural pacing modality still conditioned by the high percentage of non-responders and coronary sinus anatomy. Conduction system pacing (CSP)—His bundle pacing (HBP) and Left bundle branch area pacing (LBBAP)- upcomes as the physiological alternative to BVP in the quest for the optimal CRT. CSP showed promising results in terms of better electro-mechanical ventricular synchronization compared to BVP. However, only a few randomized control trials are currently available, and technical challenges, along with the lack of information on long-term clinical outcomes, limit the establishment of a primary role for CSP over conventional BVP in CRT candidates. This review provides a comprehensive literature revision of potential applications of CSP for CRT in diverse clinical scenarios, underlining the current controversies and prospects of this technique.

## Introduction

Cardiac resynchronization therapy (CRT) by means of biventricular pacing (BVP) is the mainstay treatment on top of guideline-directed medical therapy for patients with heart failure (HF) with reduced ejection fraction, especially when coupled with electro-mechanical ventricular dyssynchrony determined by left bundle branch block (LBBB) (Glikson et al., 2021). Several randomized clinical trials (RCTs) have demonstrated that BVP improves clinical outcomes and long-term survival, halting adverse cardiac remodeling and reducing HF hospitalizations (Mehta et al., 2021). Nonetheless, BVP is a non-physiological pacing modality that restores ventricular synchronization through the fusion of two wavefronts from the left ventricular (LV) epicardium and right ventricular (RV) endocardium (Ploux et al., 2015). Consequently, BVP produces only modest ventricular resynchronization with a relatively small reduction in QRS duration and LV activation time (LVAT) (Ploux et al., 2015). In addition, unfavorable coronary sinus venous anatomy, along with high thresholds at implantation with the potential risk of collateral phrenic nerve stimulation, further challenges the procedural success and lessen the response to this therapy (Varma et al., 2019). Indeed, up to one-third of patients undergoing BVP may not derive complete clinical benefit despite successful implantation.

In this complex scenario, conduction system pacing (CSP)—His bundle pacing (HBP) and Left bundle branch area pacing (LBBAP)- stimulating the specialized His-Purkinje (HP) system can reproduce the physiological and evolutionary form of intrinsic ventricular electro-mechanical coordination and may represent a valuable alternative in the quest of the optimal cardiac resynchronization therapy (Padala and Ellenbogen, 2020; Upadhyay et al., 2020).

Selective and non-selective HBP (S- and NS-HBP, respectively) are the terminologies used to describe the capture of the His bundle (HB): S-HBP results in the capture of the His bundle alone without myocardial capture, whereas in NS-HBP, in addition to HB there is the capture of surrounding septal myocardium.

In LBBAP, the different grades of penetrance of the pacing lead within the interventricular septum (IVS) and the demonstration of the capture of the conduction system (left bundle branch) discriminate between selective or non-selective-left bundle branch pacing (S- and NS-LBBP, respectively) and left ventricular septal pacing (LVSP) where only myocardial capture of the left side of the IVS is obtained.

**FIGURE 1:**
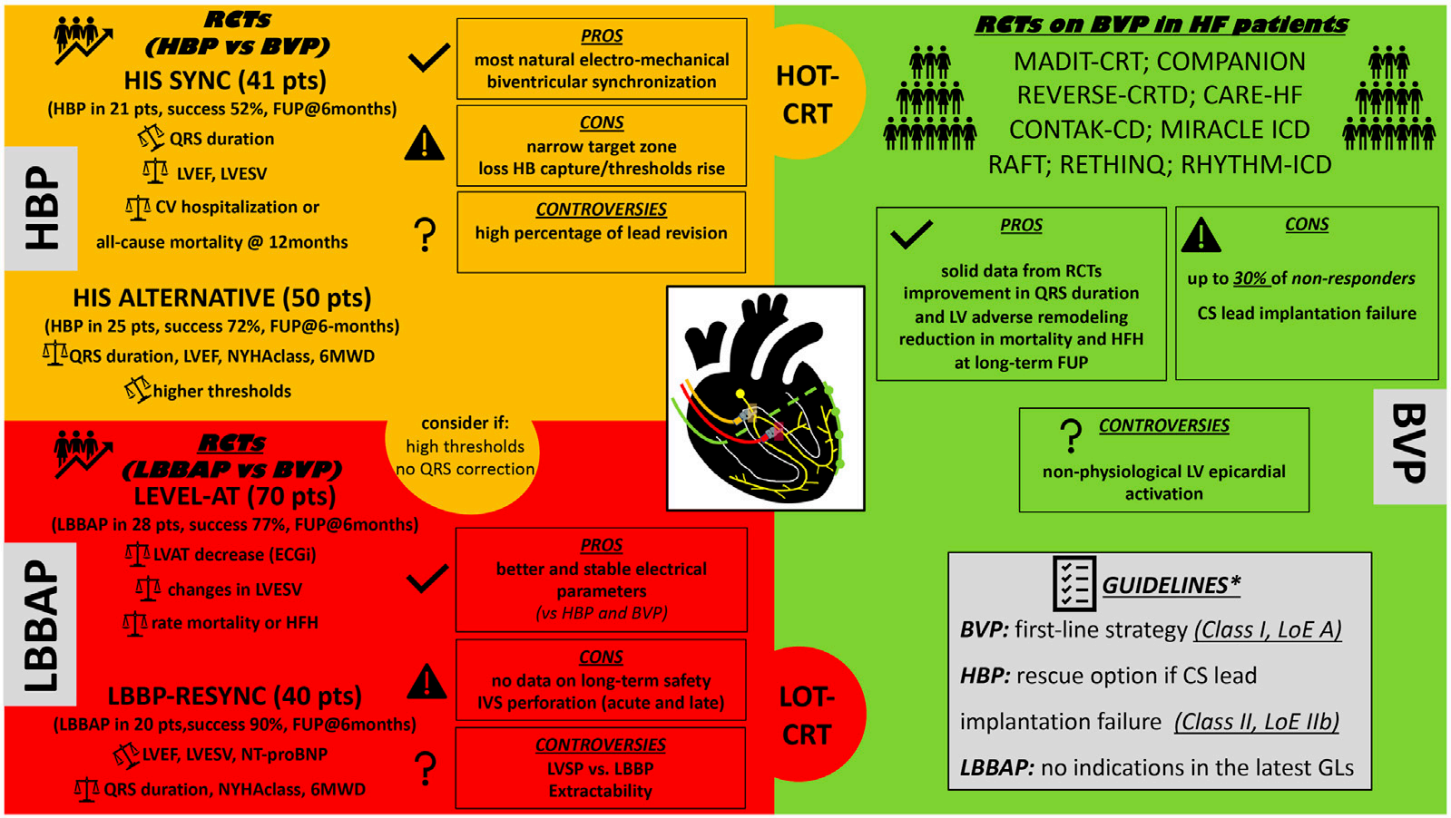
Proposed schematic “puzzle” resuming the available CRT techniques to perform cardiac resynchronization therapy. HBP, His bundle pacing; RCTs, randomized control trials; BVP, biventricular pacing; LVEF, left ventricle ejection fraction; LVESV, left ventricle end systolic volume; HB, His Bundle; NYHA, New York Heart Association functional class; 6MWD, 6-minutes walking distance; CV, cardiovascular; FUP, follow-up; LBBAP, left bundle branch area pacing; LVAT, left ventricle activation time; CRT, cardiac resynchronization therapy; HOT-CRT, His optimized CRT; LOT-CRT, left bundle branch pacing optimized CRT; HFH, heart failure hospitalizations; LVSP, left ventricle septal pacing; IVS, interventricular septum; RCTs, randomized control trials; CS, coronary sinus; LV, left ventricle; LoE, level of evidence; GLs, guidelines. *2021 ESC Guidelines on cardiac pacing and cardiac resynchronization therapy. Eur Heart J. 2021; 42 (35): 3427-520.

Despite the promising results of CSP in observational studies, only few pilot RCTs are currently available, and technical obstacles, along with the lack of information on safety issues and long-term clinical outcomes, limit the establishment of a primary role for CSP over conventional BVP in CRT candidates. This review provides a comprehensive literature revision of potential applications of CSP for CRT, highlighting current controversies and future perspectives (Figure 1).

## Electromechanical implications of CSP for CRT

The electrical activation pattern of the human heart was first described in the late 1960s (Durrer et al., 1970).

The ventricular electrical activation starts in endocardial areas on the left surface of the interventricular septum (i.e., at the left bundle branch level), then proceeds towards the apicobasal direction of the ventricles through the presence of Purkinje system terminations within the endocardium (Durrer et al., 1970).

Cardiac resynchronization therapy’s paradigm is to correct the delayed activation of the left ventricular (LV) free wall as induced by abnormalities within the HPS, namely left bundle branch block (LBBB). Previous studies showed that QRS narrowing after BVP implantation is prognostically essential mainly in patients with LBBB, and the degree of QRS duration narrowing strongly predicts clinical outcomes (Jastrzębski et al., 2018).

Several mechanisms have been postulated by which CSP normalizes the QRS duration in patients with BBBs. In the functional longitudinal dissociation concept, it is hypothesized that BBBs are likely secondary to a delay/block within the fibers in the His bundle (HB) already predestined to the right or left bundle branch and, therefore, can be customarily recruited pacing the His bundle (Narula, 1977).

Upadhyay and colleagues also proved the evidence for the proximal disease by detailed intracardiac mapping of the LV septum in 72 patients with LBBB(10). The authors reported a complete conduction block in 64% of the patients (72% at left His and 28% at proximal LBB) corrigible by pacing distally to the site of the block. Conversely, in the remaining 36% of the cases they reported absence of conduction block and intact Purkinje activation. In this latter situation HBP led to inefficient QRS correction as the delayed LV activation was likely to be due to distal conduction myocardial tissue disease (commonly observed in intraventricular conduction delay - IVCD). Other supposed mechanisms of BBBs correction with CSP may include the virtual electrode effect, transverse connections between the bundles, and the retrograde activation of HB and the right bundle branch (RBB), especially with LBBAP (11).

HBP, with either selective or non-selective capture, appears as the most physiological pacing modality that preserves or restores electrical and mechanical synchrony by simultaneously activating both ventricles. LBBAP, on the other hand, by direct capturing the LBB and manifesting electrocardiographically as an incomplete RBBB with a relatively narrow QRS duration, preserves or restores mainly the physiological activation of the LV (5). However, the clinical impact of a delayed RV activation deriving from LBBAP is still unknown, and whether atrioventricular (AV) delay adjustments or bipolar stimulation (anodal capture) could mitigate it through the contemporary right septal activation need further investigations (Padala and Ellenbogen, 2020). Nevertheless, limited studies showed comparable LV mechanical synchrony between HBP and LBBAP, but further insights are indispensable for an exhaustive understanding of the electromechanical effects of CSP and their implications for CRT (Curila et al., 2020; Curila et al., 2021; Rijks et al., 2022).

## HBP for CRT in LBBB

CRT with BVP is currently recommended with class I indication by guidelines for symptomatic heart failure patients with QRS duration>150 msec (LBBB morphology), and an LVEF <35% despite optimized medical treatment as BVP has solidly demonstrated in RCTs to improve long-term survival and HF hospitalizations (Glikson et al., 2021). However, the heterogenous effects of BVP, the unphysiological ventricular activation, and the need for three leads shed light on HBP as a physiological alternative with the potential for a first-line strategy to achieve CRT. There is already a growing body of evidence that HBP-CRT is feasible and, when successful, translates into improved LVEF and New York Heart Association (NYHA) functional class (Qi et al., 2020).

The feasibility of CRT with HBP was first described by Barba and colleagues in 2013. They selected patients from a population with refractory HF in whom LV stimulation *via* the coronary sinus was not achievable. Direct His-bundle pacing corrected basal conduction disturbances in 13 of the 16 patients (81%) selected. In four patients in whom HBP was attempted, the electrode was not successfully fixed. In the nine remaining patients (9/13, 69%), a definitive resynchronization by HBP was achieved, with consequent improvement in functional class and parameters of LV function as assessed by echocardiography (Barba-Pichardo et al., 2012).

Subsequently, in 2015, Lustgarten and colleagues performed a crossover study comparing HBP *versus* BVP in 29 CRT patients, with successful resynchronization in 21 cases (72%) (Lustgarten et al., 2015). After a successful implant, patients were randomized in a single patient-blinded fashion to either HBP or BVP. After 6 months, patients were crossed over to the other pacing modality and followed for another 6 months. Among the 12 patients who completed the crossover analysis, patients demonstrated significant improvements in LVEF, NYHA functional status, and 6-min walk distance with both HBP and BVP(17).

In a retrospective multi-center study of HBP for CRT in 106 patients performed as a primary (73 patients) or rescue (33 patients) strategy, Sharma and colleagues reported an overall success rate of 90%. During a mean follow-up of 14 months, in both groups demonstrated a significant narrowing of QRS durations, an increase in LVEF, and improvement in NYHA functional class (Sharma et al., 2018a).

So far, only the results of two small pilot RCTs comparing HBP *versus* BVP are available (Upadhyay et al., 2019b; Vinther et al., 2021). The His-SYNC study included a total of 41 patients meeting standard indications for CRT from 7 centers: 21 were randomized to the HBP-CRT group and 20 to the BVP-CRT group, with 6 months of follow-up. The findings were limited by the inclusion of patients with IVCD QRS patterns that do not always respond to HBP and the significant cross-over between groups, 48% in the HBP and 26% in the BVP group. Although the crossover rate was high, the results of the Intention to treat analysis (ITT) showed that QRS duration was significantly shorter in those that received HBP-CRT compared to those that received BVP-CRT (125 ± 22 m *versus* 164 ± 25 m; *p* < 0.001). The median LVEF improvement in HBP was higher but not significantly different from BVP (+7.2% [5.0%–16.9%] *versus* +5.9% [1.5%–11.3%], *p* = 0.17) (Upadhyay et al., 2019b). No significant differences in cardiovascular hospitalization or mortality were observed at 12 months between the groups.

The His-Alternative study showed that in HF patients with LBBB, HBP provided similar clinical and physical improvement compared with BVP at the expense of higher pacing thresholds. In this study, 51 patients were randomized 1:1 to HBP-CRT or BVP-CRT and followed for 6 months. His-corrective pacing was achieved in 72% of the patients in the HBP group. At ITT analysis at the 6-month follow-up, LVEF increased by 16 ± 7% in the HBP group compared with 13 ± 6% in the BVP group (non-significant). Pacing thresholds were higher for HBP compared with BVP both at implantation (1.8 ± 1.2 V vs. 1.2 ± 0.8 V; *p* < 0.01) and at 6-month follow-up (2.3 ± 1.4 V vs. 1.4 ± 0.5 V; *p* < 0.01). This study was limited by the high rate of cross-over that was mainly related to the technical impossibility of achieving the target QRS duration in the HBP group (Vinther et al., 2021).

According to the available data, the latest guidelines don’t consider HBP as a first-line strategy for CRT, but recommend HBP (class IIb) in CRT candidates in whom coronary sinus lead implantation is unsuccessful (Glikson et al., 2021). Although HBP showed a promising role in delivering CRT, larger endpoint clinical trials are indeed warranted to confirm its positive impact on long-term clinical outcomes and widen its application.

## LBBAP for CRT in LBBB

The procedural challenges faced with HBP and the evidence of a high rate of instability of pacing parameters at follow-up opened the way for LBBAP (Keene et al., 2019; Cano and Vijayaraman, 2021). The feasibility of deep septal pacing was first described by Mafi-Rad and colleagues showing acute hemodynamic benefits over RVP (Mafi-Rad et al., 2016).

In 2017, Huang and colleagues pioneered LBB pacing in a patient in whom HBP failed to correct LBBB at the highest pacing output. They were able to capture the proximal trunk of LBB at lower and stable output advancing the lead tip distally from HB towards the ventricle, obtaining the best QRS narrowing (after adjusting the AV delay), and observing a remarkable increase in LVEF at 1 year follow-up (Huang et al., 2017).

The initial results of prospective observational studies on LBBAP are encouraging, with LBBAP lead implantation success ranging from 80% to 97%, and tremendous expectations are reposted in LBBAP for achieving resynchronization therapy in patients with HF and LBBB (Huang et al., 2020; Heckman et al., 2021; Jastrzębski et al., 2022a; Grieco et al., 2022). A seminal study by Huang and colleagues reported a high LBBAP success rate (97%) as a first-line strategy among 63 patients with LBBB and non-ischemic cardiomyopathy (Huang et al., 2020). During a mean follow-up of 1 year, the LVEF increased significantly from 33 ± 8% to 55 ± 10% (*p* <.001) and there were no deaths or HF hospitalizations. Vijayaraman and colleagues, in a multicenter study, reported an 85% LBBAP success rate among 325 patients with LVEF<50% who were referred for CRT. During a mean follow-up of 6 months, there was a significant decrease in QRS duration and an improvement in clinical and echocardiographic response with LBBAP (Vijayaraman et al., 2021). Furthermore, in a multicenter international observational study, LBBAP showed to be a viable rescue alternative to BVP in 200 patients who had coronary vein (CV) lead failure or were non-responders, resulting in significant QRS narrowing from 170 ± 28 m to 139 ± 25 m (*p* <.001) and LVEF improvement from 29% ± 10% at baseline to 40% ± 12% (*p* <.001) (Vijayaraman et al., 2022a).

To date, only a small prospective randomized trial has been published on LBBAP compared to BVP in patients with HF (non-ischemic cardiomyopathy-NICM) and LBBB with a 6-month follow-up (Wang et al., 2022). This study included 40 consecutive patients (20 males, mean age 63.7 years, LVEF 29.7% ± 5.6%). Despite crossovers occurring in 10% of LBBP and 20% of BVP, ITT analysis showed significantly higher LVEF improvement at 6 months after LBBP than BVP (mean difference: 5.6%; 95% CI: 0.3–10.9; *p* = 0.039). LBBP also appeared to have greater reductions in LV end-systolic volume (−24.97 ml; 95% CI: −49.58 to −0.36 ml) and NT-proBNP (−1,071.80 pg/ml; 95% CI: −2,099.40 to −44.20 pg/ml) than BVP whereas comparable changes in NYHA functional class, 6-min walk distance, QRS duration, and rates of response were reported (Wang et al., 2022).

A recent study compared in a crossover fashion, instead, the acute improvement of electrico-mechanical synchrony, and hemodynamics between LBBAP and BVP in 21 patients with HF (predominantly NICM- 90% of the cases) and LBBAP (Liang et al., 2022). LBBAP achieved a larger reduction in QRS duration [−11 m (95% CI, −17 to −4 m); *p* = 0.003], QRS area [−85 μVs (95% CI, −113 to −56 μVs); *p* < 0.001], and significantly higher increase in dP/dtmax [6% (95% CI, 2%–9%); *p* = 0.002] compared to BVP.

In the published results from a large multicenter cohort of patients with HFrEF requiring CRT, CSP (either HBP or LBBAP) was associated with a significant reduction in the composite outcomes of all-cause mortality or HF hospitalizations compared to conventional BVP with a difference more pronounced in the subgroup of patients with LBBB (Vijayaraman et al., 2022b). No direct comparison between LBBAP and HBP is reported in this study, although previous network meta-analysis, found that both LBBAP and HBP resulted in a greater improvement and narrower QRS duration than BVP but with the advantages of significantly lower pacing thresholds with LBBAP (Hua et al., 2022).

In a recent non-inferiority small RCT, 70 patients with CSP indication were randomized 1:1 to CSP or BVP, and followed up for 6 months. HBP was pursued in 7 of 35 (20%) patients allocated to CSP, with an implant success in 4 of 7 (57%) patients. In 28 of 35 patients, LBBP was pursued with an implant success in 23 of 28 (82%) patients. Eight (23%) patients crossed over from CSP to BVP; 2 patients (6%) crossed over from BVP to CSP. A similar decrease in LVAT - evaluated with the non-invasive 3-dimensional mapping system (ECGi) - was achieved by CSP and BiVP (−28 ± 26 m vs. −21 ± 20 m; *p* < 0.001 for non-inferiority). Both groups showed a similar change in left ventricular end-systolic volume (−37 ± 59 ml CSP vs. −30 ± 41 ml BVP; *p* = 0.04 for non-inferiority) and similar rates of mortality or heart failure hospitalizations (2.9% CSP vs. 11.4% BVP, *p* = 0.002 for non-inferiority) (Pujol-Lopez et al., 2022).

Current international guidelines do not reserve indications for LBBAP in routine clinical practice but the effect of LBBAP on electro-mechanical parameters seems impressively promising.

Further confirmation of LBBAP impact on clinical endpoints at long-term is deeply necessary from larger RCTs to favor the adoption of this technique over BVP in patients undergoing CRT.

## Limitations and controversies

The incremental diffusion of CSP is laden by troubleshooting issues for HBP and concerns about long-term performances for LBBAP (Padala and Ellenbogen, 2020; Upadhyay et al., 2020). Since the HB is located in a strict zone encased in electrically inert fibrous tissue, HBP leads typically have a low R-wave amplitude that may result in over-sensing atrial or His signals and under-sensing of ventricular signals (Padala and Ellenbogen, 2020; Upadhyay et al., 2020). The higher HBP capture thresholds at implantation and during follow-up may predispose to premature battery depletion and repeated generator replacements with relative risks (Padala and Ellenbogen, 2020; Upadhyay et al., 2020). Moreover, the unpredictable, delayed rise in HBP capture thresholds is a significant concern, resulting in high lead revision rates, described in up to 11% of the cases (Teigeler et al., 2021).

Despite LBBAP advantages (e.g., more stable position and better pacing parameters), some safety issues are still unknown as it has been widely used only since 2017 (Padala and Ellenbogen, 2020). Some complications, such as acute and late perforation in the LV, can occur from the pacing lead screwed deep within the septum (Zhang et al., 2019). While acute perforations without adverse hemodynamic sequelae seem easily solvable with lead repositioning, the potential thrombogenic risk, if the LBBP lead tip remains chronically exposed in the LV chamber, needs to be ascertained (Zhang et al., 2019). Multiple attempts at lead placement or manipulation within the septum may also lead to myocardial damage or potential injury of septal branch arteries. Not least, the feasibility of LBBAP lead extraction from the deep septal position is still under-investigated.

In addition to strengths and limitations, CSP brings some controversies that need further explanation. For example, LVSP showed better interventricular synchrony but prolonged LV lateral wall depolarization than LBBP in bradycardia patients and comparable short-term hemodynamic and electro-mechanical improvements with BVP and HBP (Curila et al., 2021; Rijks et al., 2022). Since LBBP requires additional electrophysiological maneuvers and surrogated criteria to assess LBB capture, it should be tested whether LVSP alone could improve the clinical outcomes of patient candidates for CRT (Rijks et al., 2022).

Finally, as most of the studies on CSP for CRT were conducted in patients with non-ischemic cardiomyopathy, its generalizability should be extensively validated also in patients with ischemic substrates, genetic or acquired cardiomyopathies (e.g., cardiac sarcoidosis) where the necrotic scar and the fibrotic process may involve the target zone of CSP lead placement.

## CSP-CRT beyond LBBB and future perspectives

Several conditions beyond LBBB can lead to cardiac dyssynchrony and may be targeted by CRT through physiological pacing.

### RBBB/IVCD

It has been demonstrated that BVP-CRT is less effective in patients with RBBB since conventional LV pacing from a coronary sinus vein is not presumed to correct the delayed activation of the RV.

Conversely, both HBP and LBBAP overcame the abovementioned limitations, conferred a significant QRS narrowing, and improved LVEF and NYHA functional class in this setting (Sharma et al., 2018b; Vijayaraman et al., 2022c). Moreover, in the case of IVCD, where conduction system alterations coexist with intramyocardial tissue delay, a more completed resynchronization resulted achievable from combining pacing the His Purkinje system with epicardial pacing of LV through BVP. Indeed, a significant clinical, electrocardiographic, and echocardiographic improvement has been observed in preliminary studies with His-optimized CRT (HOT-CRT) and LBBAP-optimized CRT (LOT-CRT) (Vijayaraman et al., 2019; Zweerink et al., 2021; Jastrzębski et al., 2022b).

### Treatment and prevention of pacing-induced cardiomyopathy (PICM)

It is renowned that conventional right ventricular pacing (RVP) induces an unnatural ventricular activation sequence dependent on slow conduction through the myocardial tissue rather than the specialized electrical system, portending to the so-called pacing-induced cardiomyopathy (PICM): a dyssynchronous ventricular activation with the consequent risk of systolic and diastolic dysfunction. However, upgrading from RVP to CSP, either with HBP or LBBAP, showed significant QRS width reduction and improvement in LVEF, suggesting that electrical and structural changes induced by chronic RVP may be reversed effectively with the adoption of CSP (Shan et al., 2018; Ye et al., 2021; Kaza et al., 2022). Moreover, especially in pacemaker candidates with expected high ventricular pacing burden (>20%), delivering more physiological ventricular activation with CSP prevented the occurrence of PICM and demonstrated a significant reduction in death, HF hospitalizations, or the need to upgrade to BVP when compared with RVP (Abdelrahman et al., 2018; Sharma et al., 2022).

### Pace and ablate

AV junction ablation (AVJA) aims to render atrial fibrillation patients pacing dependent to optimize ventricular rate control and response to CRT. Still, at the same time, it could favor the occurrence of PICM, especially in those with pre-existing impaired LV function. This seems more likely with chronic RVP, even though BVP also resulted in dyssynchrony in patients with normal QRS at baseline. The potential advantage of CSP is that it can preserve ventricular synchronization in patients undergoing AVJA that are vulnerable to PICM. Preliminary studies showed that AVJA is technically feasible in the presence of a CSP lead, portending significant improvement in QRS duration, LVEF, and better clinical outcomes (reduction of death and HF hospitalizations) compared with RVP and BVP (Vijayaraman et al., 2022d). Recently, in a multicenter, prospective, randomized crossover trial, enrolling 50 patients undergoing AVJA, HBP delivered a modest but significant improvement in LVEF in patients with persistent AF, impaired left ventricular function (LVEF≤40%), and narrow QRS duration, compared with BVP (Huang et al., 2022). Noteworthy, in an observational study, AVJA in the presence of an LBBAP lead was associated with a higher success rate and fewer acute and chronic lead-related complications compared to AVJA in the presence of an HBP lead (Pillai et al., 2022).

### Future perspectives

CSP, in particular LBBAP, is currently performed using leads not designed and conceived initially with this scope (De Pooter et al., 2022); therefore, refinements in tools and technicalities of the procedure are necessary to improve the overall success rate and long-term safety profile.

Moreover, in the CROSS-LEFT pilot study in patient candidates for CRT, an LBBAP lead connected to a DF-1 dual-chamber implantable cardioverter-defibrillator provided safe ventricular arrhythmia sensing and efficient electro-mechanical resynchronization (Clementy et al., 2022). With the addition of defibrillation capability, this could prospect the chance to achieve cardiac resynchronization and anti-tachycardia therapies using a unique LBBAP lead in the future.

## Conclusions

The up-to-date clinical evidence for BVP outnumbers that for CSP; therefore, the results of larger prospective RCTs with long-term follow-up are awaited to establish a definitive principal role for CSP *in lieu* of BVP in patients requiring CRT.

Meanwhile, a tailored analysis of the underlying ventricular desynchronization patterns and optimal patient selection may help identify HF patients amenable to benefit most from CSP-CRT as an alternative or in combination with BVP.
