# Supplementary material for: Conduction system pacing for cardiac resynchronization therapy: State of the art, current controversies, and future perspectives
Source: Front Physiol. 2023 Jan 13;14:1124195. doi: 10.3389/fphys.2023.1124195 (PMC9880410; doi:10.3389/fphys.2023.1124195)
Supplement: Supplementary file 1 [file Table1.docx]

**Supplementary Table.** Summary of published trials on His bundle pacing (HBP) and Left bundle branch area pacing (LBBAP) for CRT indication with echocardiographic and/or clinical follow-up.

| **Study** | **Design** | **Inclusion criteria** | **n**  **(% success)** | **Follow-up** | **Findings and comments** |
| --- | --- | --- | --- | --- | --- |
| **HBP in lieu of BVP** | | | | | |
| **Barba-Pichardo et al. 2013** *(1)* | Observational | Failed LV lead implantation  LBBB corrected by temporary HBP | 13  (9/13, 69%) | 2.6±1.8 years | Improvement in LVEF and LV dimensions reduction in NYHA class compared to baseline.  Stylet-driven leads used without guiding catheter. |
| **Lustgarten et al. 2015***(2)* | Randomized Crossover  Blinded echo  HBP vs BVP  Two-centre | CRT indication  QRS>130ms  QRS narrowing with HBP | 29  (21/29, 72%) | 2x6 months | No difference in LVEF, QOL, NYHA, 6MWT between HBP and BVP. |
| **Ajijola et al. 2017***(3)* | Observational Two-centre | CRT indication | 21  (16/21, 76%) | Median 1 year | Significant improvement in LVEF and LV dimensions, NYHA class compared to baseline. Super-response in 3 (19%) patients. |
| **Sharma et al. 2018** *(4)* | Observational  Retrospective  Multicentre | CRT indication (primary or “rescue” HBP) | 106 (95/106, 90%) | 14.4±15 months | Significant improvement in LVEF and NYHA class compared to baseline. Super-response in 27 (39%) patients. |
| **Shan et al. 2018** *(5)* | Observational  Single centre | LVEF<50%  RV pacing or BVP non-response | 18  (16/18, 89%) | 36±14 months | Significant improvement in LVEF, LV dimensions, mitral regurgitation, NYHA class compared to baseline. |
| **Huang et al. 2019***(6)* | Observational  Single centre  Prospective | LBBB NYHA II-IV CRT or pacing indication | 74  (56/74, 76%) | Median 37 months | Similar improvement in LVEF, LVESV and NYHA at 1 year between HBP group compared to BVP group.  Significant improvement in LVEF and LVESV compared to baseline with HBP over 3 year follow-up |
| **Upadhyay et al. 2019**  *(7,8)* | Multicentre Parallel Single-blind  (HBP vs BVP) | CRT indication | 21 vs 20  (11/21, 52%) | 12 months | Greater improvement in QRS duration with HBP (significant as treated, trend by ITT), and rend in greater improvement in LVEF. No difference in HF hospitalization/mortality.  Crossover 46% from HBP to BVP and 26% from BVP to HBP. |
| **Vinther**  **2021**  *(9)* | Single-center, prospective, randomized control trial  (HBP vs BVP) | CRT indication | 25 vs 25  (18/25, 72%) | 6 months | HBP provided similar clinical and physical improvement compared to BVP at the expenses of higher thresholds at implantation and follow-up.  Crossover 28% from HBP to BVP and 4% from BVP to HBP. |
| **LBBAP in lieu of BVP** | | | | | |
| **Li *et al.* 2020**  *(10)* | Prospective, multicenter,  observational | HF symptoms, LVEF ≤ 35% with LBBB | 27 vs 54  (27/37, 73% success) | 6 months | NYHA: 1.5 ± 0.5 vs. 2.3 ± 0.7  Echocardiographic response: 88.9% vs. 66.7%  clinical response: 96.3% vs. 75.9% |
| **Wang *et al.***  **2020***(11)* | case–control study | HF, LBBB with QRSd>140msec(men) and >130msec (women), LVEF≤35%, NYHA II-IV | 10 vs 30  (10/10, 100% success) | 6 months | LVEF: 45.66 ± 9.22% vs. 39.35 ± 12.29%  NYHA: 1.50 ±0.55 vs. 1.97 ± 0.61 |
| **Guo et al**  **2020** *(12)* | Prospective, observational | HF, LBBB with QRSd>140msec(men) and >130msec (women), LVEF≤35%, NYHA II-IV | 21 VS 21  (LBBAP 21/24, 87% success) | 6 months | LVEF: 50.9 ± 10.7% vs. 44.4 ± 13.3%  NYHA: 1.3 ± 0.9 vs. 1.5 ± 0.7 |
| **Wu et al**  **2021** *(13)* | Prospective, non-randomized, single-center | LBBB with LVEF≤40% | 32 vs. 54 ( 32/32, 100%success) | 12 months | LVEF: 54.4±9.8% vs. 46.5±16.9%  NYHA: 1.3 ±0.5 vs. 1.9 ± 0.9 |
| **Zu et al**  **2021** *(14)* | Observational | HF with LBBB (excluded ischemic DCM) | 13 vs. 19  (13/13, 100%success) | 12 months | LVEF: 48.92 ± 8.06% vs. 42.53 ± 4.89% |
| **Chen et al**  **2021***(15)* | Prospective multicenter, observational | HF with typical LBBB, LVEF≤35%, | 49 vs. 51  (49/50, 98% success) | 12 months | LVEF: 49.10 ± 10.43% vs. 43.62 ± 11.33%  NYHA (percentage of III–IV): 4.08%vs. 19.61% |
| **Liu et al**  **2021** *(16)* | Prospective multicenter, cohort study | HF with LBBB, LVEF≤35%, QRS> 130 msec | 27 vs. 35  (27/34, 79% success) | 6 months | LVEF: 47.1 ± 8.3% vs. 43.1± 11.0%  NYHA: 1.6 ± 0.6 vs. 2.2±0.8 |
| **Wang et al 2022** *(17)* | Prospective, randomized trial | HF with LBBB (Strauss definition), LVEF≤40%, QRS>130msec (women) and QRS>140 msec (men), NYHA II-IV. | 22 vs. 18  (22/24, 92% success) | 6 months | LVEF: 49.4±13.2% vs. 46.5± 9.4%  ΔNYHA: 1.22 ±0.11 vs. 1.10 ± 0.11  Δ6-minute walk distance: 100.69±14.14m vs. 80.56±15.92m  ΔNT-proBNP: 1768.36±217.91pg/ml vs.1181.05 ± 216.75 pg/ml  Crossover 10% from LBBP to BVP and 20% from BVP to LBBP. |
| **His optimized CRT (HOT-CRT)** | | | | | |
| **Vijayaraman et al.**  **2019** *(18)* | Observational  Retrospective  Multicentre | LBBB/IVCD with incomplete correction by HBP  NYHA III/IV LVEF<35% | 27  (25/27, 93% success) | 14±10 months | Significant improvement in LVEF and NYHA class compared to baseline. |
| **Deshmukh et al**  **2020***(19)* | Retrospective, single center | CRT indication in whom HBP not resulted in resynchronization | 21  (21/21, 100%) | 32 months | Improvement in NYHA functional class (from 3 to 2)  HOT-CRT resulted in superior acute electrical synchrony |
| **Zweerink et al 2021** *(20)* | Prospective, single center, observational | CRT indication | 19 | NA | HOT-CRT resulted in acute improvement in electrical synchrony compared to BVP and HBP  HOT-CRT reduced LVAT by 21% comparared to HBP |
| **Left bundle branch pacing optimized CRT (LOT-CRT)** | | | | | |
| **Jastrzębski et al 2021** *(21)* | Prospective  Observational  Multicentre | CRT indication or non-responders to BVP | 112  (91/112, 81% success) | 3 months | greater electrical synchrony with LOT-CRT compared to BVP and LBBAP  NYHA functional class 2.9->1.9 |

1. Barba-Pichardo R, Manovel Sanchez A, Fernandez-Gomez JM, Morina-Vazquez P, Venegas-Gamero J, Herrera-Carranza M. Ventricular resynchronization therapy by direct His-bundle pacing using an internal cardioverter defibrillator. Europace 2013; 15: 83-88.
2. Lustgarten DL, Crespo EM, Arkhipova-Jenkins I, Lobel R, Winget J, Koehler J, et al. His-bundle pacing versus biventricular pacing in cardiac resynchronization therapy patients: A crossover design comparison. Heart Rhythm 2015; 12: 1548-1557.
3. Ajijola OA, Upadhyay GA, Macias C, Shivkumar K, Tung R. Permanent His-bundle pacing for cardiac resynchronization therapy: Initial feasibility study in lieu of left ventricular lead. Heart Rhythm 2017.
4. Sharma PS, Dandamudi G, Herweg B, Wilson D, Singh R, Naperkowski A, et al. Permanent His-bundle pacing as an alternative to biventricular pacing for cardiac resynchronization therapy: A multicenter experience. Heart Rhythm 2018; 15: 413-420.
5. Shan P, Su L, Zhou X, Wu S, Xu L, Xiao F, et al. Beneficial effects of upgrading to His bundle pacing in chronically paced patients with left ventricular ejection fraction <50. Heart Rhythm 2018; 15: 405-412.
6. Huang W, Su L, Wu S, Xu L, Xiao F, Zhou X, et al. Long-term outcomes of His bundle pacing in patients with heart failure with left bundle branch block. Heart 2019; 105: 137-143.
7. Upadhyay GA, Vijayaraman P, Nayak HM, Verma N, Dandamudi G, Sharma PS, et al. On-treatment comparison between corrective His bundle pacing and biventricular pacing for cardiac resynchronization: A secondary analysis of His-SYNC. Heart rhythm 2019.
8. Upadhyay GA, Vijayaraman P, Nayak HM, Verma N, Dandamudi G, Sharma PS, et al. His Corrective Pacing or Biventricular Pacing for Cardiac Resynchronization in Heart Failure. Journal of the American College of Cardiology 2019; 74: 157-159.
9. Vinther M, Risum N, Svendsen JH, Møgelvang R, Philbert BT. A Randomized Trial of His Pacing Versus Biventricular Pacing in Symptomatic HF Patients With Left Bundle Branch Block (His-Alternative). JACC Clin Electrophysiol. 2021 Nov;7(11):1422-1432.
10. Li X, Qiu C, Xie R,Ma W, Wang Z, Li H, et al. Left bundle branch area pacing delivery of cardiac resynchronization therapy and comparison with biventricular pacing. ESC Heart Fail. (2020) 7:1711–22. doi: 10.1002/ehf2.12731
11. Wang Y, Gu K, Qian Z, Hou X, Chen X, Qiu Y, et al. The efficacy of left bundle branch area pacing compared with biventricular pacing in patients with heart failure: a matched case-control study. J Cardiovasc Electrophysiol. (2020) 31:2068–77. doi: 10.1111/jce.14628
12. Guo J, Li L, Xiao G, Ye T, Huang X, Meng F, et al. Remarkable response to cardiac resynchronization therapy via left bundle branch pacing in patients with true left bundle branch block. Clin Cardiol. (2020) 43:1460–8. doi: 10.1002/clc.
13. Wu S, Su L, Vijayaraman P, Zheng R, Cai M, Xu L, et al. Left bundle branch pacing for cardiac resynchronization therapy: nonrandomized on-treatment comparison with his bundle pacing and biventricular pacing. Can J Cardiol. (2021) 37:319–28. doi: 10.1016/j.cjca.2020.04.037
14. Zu L, Wang Z, Hang F, Jiang Y, Wang X, Cheng L, et al. Cardiac resynchronization performed by Lbbap-Crt in patients with cardiac insufficiency and left bundle branch block. Ann Noninvasive Electrocardiol. (2021) 26:e12898. doi: 10.1111/anec.12898
15. Chen X, Ye Y, Wang Z, Jin Q, Qiu Z, Wang J, et al. Cardiac resynchronization therapy via left bundle branch pacing vs. optimized biventricular pacing with adaptive algorithm in heart failure with left bundle branch block: a prospective, multi-centre, observational study. Europace. (2021) 24:807–16. doi: 10.1093/ europace/euab249
16. Liu W, Hu C, Wang Y, Cheng Y, Zhao Y, Liu Y, et al. Mechanical synchrony and myocardial work in heart failure patients with left bundle branch area pacing and comparison with biventricular pacing. Front Cardiovasc Med. (2021) 8:727611. doi: 10.3389/fcvm.2021.727611
17. Wang Y, Zhu H, Hou X, Wang Z, Zou F, Qian Z, et al. Randomized trial of left bundle branch vs biventricular pacing for cardiac resynchronization therapy. J Am Coll Cardiol. (2022) 80:1205–16. doi: 10.1016/j.jacc.2022.07.019
18. Vijayaraman P, Herweg B, Ellenbogen KA, Gajek J. His-Optimized Cardiac Resynchronization Therapy to Maximize Electrical Resynchronization. Circ Arrhythm Electrophysiol 2019; 12: e006934.
19. Deshmukh A, Sattur S, Bechtol T, et al. Sequential His bundle and left ventricular pacing for cardiac resynchronization. J Cardiovasc Electrophysiol 2020; 31(9):2448–54.
20. Zweerink A, Zubarev S, Bakelants E, et al. His-optimized cardiac resynchronization therapy with ventricular fusion pacing for electrical resynchronization in heart failure. JACC Clin Electrophysiol 2021;7(7): 881–92.
21. Jastrzębski M, Moskal P, Huybrechts W, Curila K, Sreekumar P, Rademakers LM, Ponnusamy SS, Herweg B, Sharma PS, Bednarek A, Rajzer M, Vijayaraman P. Left bundle branch-optimized cardiac resynchronization therapy (LOT-CRT): Results from an international LBBAP collaborative study group. Heart Rhythm. 2022 Jan;19(1):13-21.
